# Systematic Literature Review and Meta-Analysis of Renal Function in Human Immunodeficiency Virus (HIV)-Infected Patients Treated with Atazanavir (ATV)-Based Regimens

**DOI:** 10.1371/journal.pone.0124666

**Published:** 2015-05-04

**Authors:** Sandrine Cure, Florence Bianic, Caroline Espinas, Helene Hardy, Lisa Rosenblatt, Timothy Juday

**Affiliations:** 1 Health Economic and Outcomes Research, OptumInsight, Uxbridge, United Kingdom; 2 Health Economic and Outcomes Research, OptumInsight, Paris, France; 3 Medical, Virology, Bristol-Myers Squibb, Plainsboro, New Jersey, United States of America; 4 Health Economic and Outcomes Research, Bristol-Myers Squibb, Plainsboro, New Jersey, United States of America; University of Cape Town, SOUTH AFRICA

## Abstract

Some HIV antiretroviral therapies (ART) have been associated with renal toxicities, which become of increasing concern as HIV-infected patients age and develop comorbidities. The objective of this study was to evaluate the relative impact of atazanavir (ATV)-based regimens on the renal function of adult patients with HIV. We conducted a systematic literature review by searching PubMed, EMBASE, Cochrane library, and the CRD from 2000 until March 2013. Major HIV-related conferences occurring in the past two years were also searched. All randomized clinical trials and large cohort studies assessing renal function in treatment-naïve and/or treatment-experienced HIV patients on ATV-based regimens were included. Fixed-effect mixed-treatment network analyses were carried out on the most frequently reported renal outcomes. 23 studies met the inclusion criteria, and change in estimated glomerular filtration rate (eGFR) from baseline to 48 weeks was identified as the main outcome. Two networks including, respectively, six studies (using the Cockcroft-Gault method) and four studies (using MDRD and CKD-EPI) were analysed. With CG network, ATV/r + TDF/FTC was associated with lower impact on the decline of eGFR than ATV/cobicistat + TDF/FTC but with higher decrease in eGFR than ATV/r + ABC/3TC (difference in mean change from baseline in eGFR repectively +3.67 and –3.89). The use of ATV/cobicistat + TDF/FTC led to a similar decline in eGFR as EVG/cobicistat/TDF/FTC. With respect to third agents combined with TDF/FTC, ATV/r had a lower increase in eGFR in comparison to EFV, and no difference was shown when compared to SQV/r and DRV/r. The effect of ATV-based regimens on renal function at 48 weeks appears similar to other ART regimens and appears to be modest regardless of boosting agent or backbone, although TDF containing backbones consistently leads to greater decline in eGFR.

## Introduction

The use of antiretroviral therapy (ART) has dramatically reduced human immunodeficiency virus (HIV)-related mortality and morbidity.[1] However, HIV-infected patients are at increased risk of premature comorbidities as a consequence of their HIV infection and the metabolic complications of ART combination; they have a nearly four-fold likelihood of developing renal disease compared to those without HIV.[2] In current clinical practice, the kidney function is assessed by the estimation of the glomerular filtration rate (eGFR) considered as the best overall indicator. The Kidney Disease Outcomes Quality Initiative (KDOQI) Practice Guidelines recommend that renal function be estimated by creatinine-based equations including the Cockcroft and Gault (CG), Modification Diet Renal Disease (MDRD) or CKD-Epidemiology (CKD-EPI).[3]

The choice of the initial ART is an extremely important decision in terms of managing HIV-infected patients. To be optimally treated, ART-naïve patients are recommended to receive a combination therapy of two NRTIs (nucleoside/nucleotide reverse transcriptase inhibitors) with one of the following ART otpions: efavirenz (EFV), a non-nucleoside reverse transcriptase inhibitor (NNRTI); darunavir (DRV) or atazanavir (ATV), protease inhibitors (PI) boosted with ritonavir (RTV); or raltegravir (RAL), dolutegravir (DTG) or Elvitegravir (EVG), integrase strand transfer inhibitors (INSTI).[4–7]

Atazanavir (ATV) (or Reyataz) is generally used in combination with the boosting agent, ritonavir (ATV/r), and is currently being developed as a fixed-dose combination with the boosting agent cobicistat (ATV/cobi). Two NRTIs—usually TDF (tenofovir) and FTC (emtricitabine)—are commonly used in combination with in an ATV-containing ART regimen.[4]

ART is known to decrease the risk of renal disease in HIV patients by 46% compared to naïve patients, however some treatments or boosted agents as part of ART regimens have been associated with renal impairment.[2] Meta-analytic results suggest that the relative risk of renal disease (i.e. defined as eGFR < 60 ml/min/1.73m^2^ for ≥ 3 months irrespective of kidney damage) is significantly increased (56%) in patients treated with TDF-containing regimens as compared to those treated with TDF-sparing regimens.[2] While the relationship between TDF and renal disease is well documented, the interactive effect on renal function of TDF use with other ART medications remains unclear. TDF has been associated with renal impairment when co-administered with some RTV-boosted PIs.[8–10] Indeed, it is postulated that RTV would block the tubular secretion of TDF. In a recent study, exposure to TDF was associated with higher risk of chronic kidney disease (CKD), proteinuria, and rapid decline in renal function, while RTV was associated with higher risk of proteinuria and ATV with higher risk rapid decline in renal function.[11] The study did not provide any insight into how the results for ATV may have been affected by its use in combination with TDF and/or RTV.

The boosting agent cobicistat, a novel pharmaco-enhancer with no antiviral activity against HIV, was developed to boost the plasma levels of elvitegravir (EVG) or PIs.[12] Cobicistat is known to inhibit tubular secretion of creatinine resulting in a modest increase in serum creatinine and a decrease in estimated glomerular filtration rate (eGFR).[13] In recent phase III clinical trials, this decline in eGFR was observed rapidly upon initiation of cobicistat when used as a booster in ART combinations followed then by a stabilization.[12;14;15]

The purpose of this study is to evaluate existing data on the impact of ATV on renal function; more specifically, its intent is to generate meta-analytic estimates of the impact that ATV-based regimens may have on renal function in HIV-infected patients, particularly in combination with or without TDF and/or RTV or cobicistat.

## Methods

### Literature review

#### Search strategy

The systematic search identified all candidate randomized controlled trials (RCTs) and large cohort studies (size above 100 patients per treatment arm) reporting outcomes on renal function of regimens containing ATV, with or without TDF and with or without boosting with ritonavir or cobicistat in treatment-naive patients with HIV. The search was conducted in March 2013 by searching Medline, EMBASE, the Cochrane library and the Centre for Review and Dissemination (CRD). The results for these searches were limited to studies conducted in humans and published in English from 2000 onwards. Major HIV-related conferences (Interscience Conference on Antimicrobial Agents and Chemotherapy or ICAAC, Infectious Diseases Society of America or IDSA, Conference on Retroviruses and Opportunistic Infections or CROI, International AIDS Society or IAS and International AIDS Conference or IAC) occurring in the past two years (2011–2013) were also searched for relevant presentations. Detailed search strategies and associated results are listed in S1 Table.

#### Inclusion/Exclusion criteria

A single reviewer assessed relevant articles for eligibility. Titles and abstracts were screened using pre-defined eligibility criteria in accordance with the PICOS (population, intervention, comparator, outcome, study design) framework (Table 1) before retrieval of the full-text articles. RCTs and large cohort studies containing one of the treatments of interest (i.e. ATV-based regimens) were included even if they did not report any safety results in the abstract. This was to avoid excluding potentially relevant articles that were unlikely to report renal safety outcomes in their abstracts when it was not the major topic of the study. Eligible studies enrolled treatment-naïve (TN) and/or treatment-experienced HIV infected adult patients receiving any of the treatments of interest. Studies were excluded if they were pharmacokinetics, pilot, animal or *in vitro* studies, narrative reviews, editorials or case report. Systematic reviews/meta-analyses and small observational studies (size less than 100 patients per treatment arm) were also excluded, but their reference list was reviewed to validate the final list of included papers.

**Table 1:** PICOS methodology.

**Population**	Adults (≥ 18 years) with a diagnosis of HIV without renal disease (defined as eGFR inferior to 60ml/min/1.73m^2^ for greater than or equal to 3 months irrespective of kidney damage) or reduced renal function
**Intervention**	**All studies assessing an ATV-containing regimen**: ATV administered with TDF; ATV administered without TDF; ATV boosted with ritonavir or cobicistat and with TDF; ATV boosted with ritonavir or cobicistat and without TDF
**Comparator**	All antiretroviral regimens included in the selected studies
**Outcome**	All renal outcomes including serum creatinine, eGFR, chronic kidney disease (CKD), acute renal failure and proteinuria
**Study design**	Randomized controlled trials (≥ Phase II) of any duration and/or large cohort studies including more than 100 patients in the ATV-based regimen arm

ATV: Atazanavir; CKD: Chronic Kidney Disease; eGFR: estimated Glomerular Filtration Rate; TDF: Tenofovir

#### Data extraction and outcomes

Data from all included studies were extracted by a single reviewer using a data extraction template developed for this purpose following the PRISMA (preferred reporting items for systematic reviews and meta-analyses) guidelines.[16] The key information recorded were author, year of publication, study design (e.g.country or region, details on study type, blinding, study duration, inclusion/exclusion criteria and treatment arms), patient characteristics (e.g. number of patients per arm, age, gender, race/ethnicity, comorbidities, CD4 cell count, smoking status and treatment history), treatment characteristics (e.g. treatment regimen, dosing) and outcomes of interest (e.g. serum or plasma creatinine, eGFR or creatinine clearance, CKD, acute renal failure and proteinuria). For each renal outcome, measured variable, measure unit, method and timing of assessment were collected. GFR can be estimated by measuring a 24 hours urine creatine clearance; however the inaccurancies associated with this method led to the development of predictive equations using serum creatinine level, age, race, sex and/or body size. The CG, MDRD and CKD EPI formulae are detailed in S2 Table.[3;17] The CG formula was developed in Caucasian inpatient setting with a large range of renal function. It uses age, sex, serum creatinine and lean body weight. In contrast to the CG formula, the MDRD and the CKD-EPI do not adjust for weight but accounts for age, sex, serum creatinine and race especially.[18] The MDRD equation was first used to study people with CKD and therefore results in imprecise measurement and underestimation of GFR at higher values. The CKD-EPI equation was developed afterwards with the goal to make it as accurate as the MDRD formula for GFR less than 60 mL/min per 1.73 m^2^ and more accurate for higher GFR.[17] Studies reporting eGFR with MDRD or CKD-EPI equations were pooled assuming that they provided equivalent measures.

The methodological quality of included RCTs and observational studies was assessed using the *Methodology Checklist* of the National Institutes Clinical Excellence (NICE) Guidelines Manual.[19]

A feasibility assessment was conducted and consisted of evaluating the heterogeneity between the outcomes reported in each study, based on the timing of assessment, measured variables, methods of measurement and units. To allow for pooling and comparison of renal safety results, only studies with the most commonly reported renal outcomes with the same assessement time and measure were selected.

The Preferred Reporting Items for Systematic Reviews and Meta-Analyses (PRISMA) guidelines were followed through all phases of the study (S3 Table).

### Statistical analysis

Treatment networks were designed for the most frequently reported outcomes, pooling results of studies with the exact same time of assessment and measure unit. Trial-specific mean eGFR changes from baseline were included in the model using a normal likelihood function. Mixed-treatment comparison (MTC) was carried out using WinBugs 1.4. in a Bayesian framework. The ATV regimen, ATV/r + TDF/FTC, was used as the common comparator. We selected to run fixed-effect models instead of random-effect models given the low number of eligible studies as we did not want to include more variability than could be explained by the studies. When at least two trials reported evidence for the same treatment comparison, the I^2^ statistic, a measure of between-study heterogeneity was estimated.

Estimates of the mean change in eGFR from baseline and standard error (SE) for each arm from each study included in the analysis were necessary to perform the MTC:

- In case the median was only available, the mean and median change from baseline were pooled assuming no skewness in the eGFR variable
- In case the standard deviation was not available, it was calculated using the interquartile (IQ) range and assuming a normal distribution.
  $${SD}_{Changefrombaseline}=\left(Q_{3}-Q_{1}\right)/\left(2\times Norm\;\text{sin}\;v\left(0.75\right)\right)$$ (1)
- In case the IQ range was not available, the SD was obtained by calculating the average of all the SDs available for the same treatment
- When no estimation was possible, the author had been contacted to provide us with the missing values
- When the standard error associated with eGFR at baseline and eGFR at time of interest were both available, the SD_change from baseline_ was calculated using the following formula assuming a coefficient of correlation of 0.70
  $$SDChangefrombaseline=\sqrt{\left({{SD}^{2}}_{Baseline}+{{SD}^{2}}_{48\;weeks}-\left(2\times corr\times {SD}_{48\;weeks}\right)\right)}$$ (2)
- For all included studies, the SE was calculated as follows:
  $$S\;\text{tan}\;dardError\left(SE\right)=SD/\sqrt{npatients}$$ (3)
  with the exception of one study where the SE was calculated using the lower and upper 95% confidence intervall (LCI and UCI):
  StandardError(SE)=(UCI−LCI)/(2×Normsinv(0.75)) (4)

The analyses were carried out on the intention-to-treat (ITT) population; when the population was not specified, it was assumed that the results were reported for the ITT population.

## Results

### Main findings from the literature review

**Fig 1:**
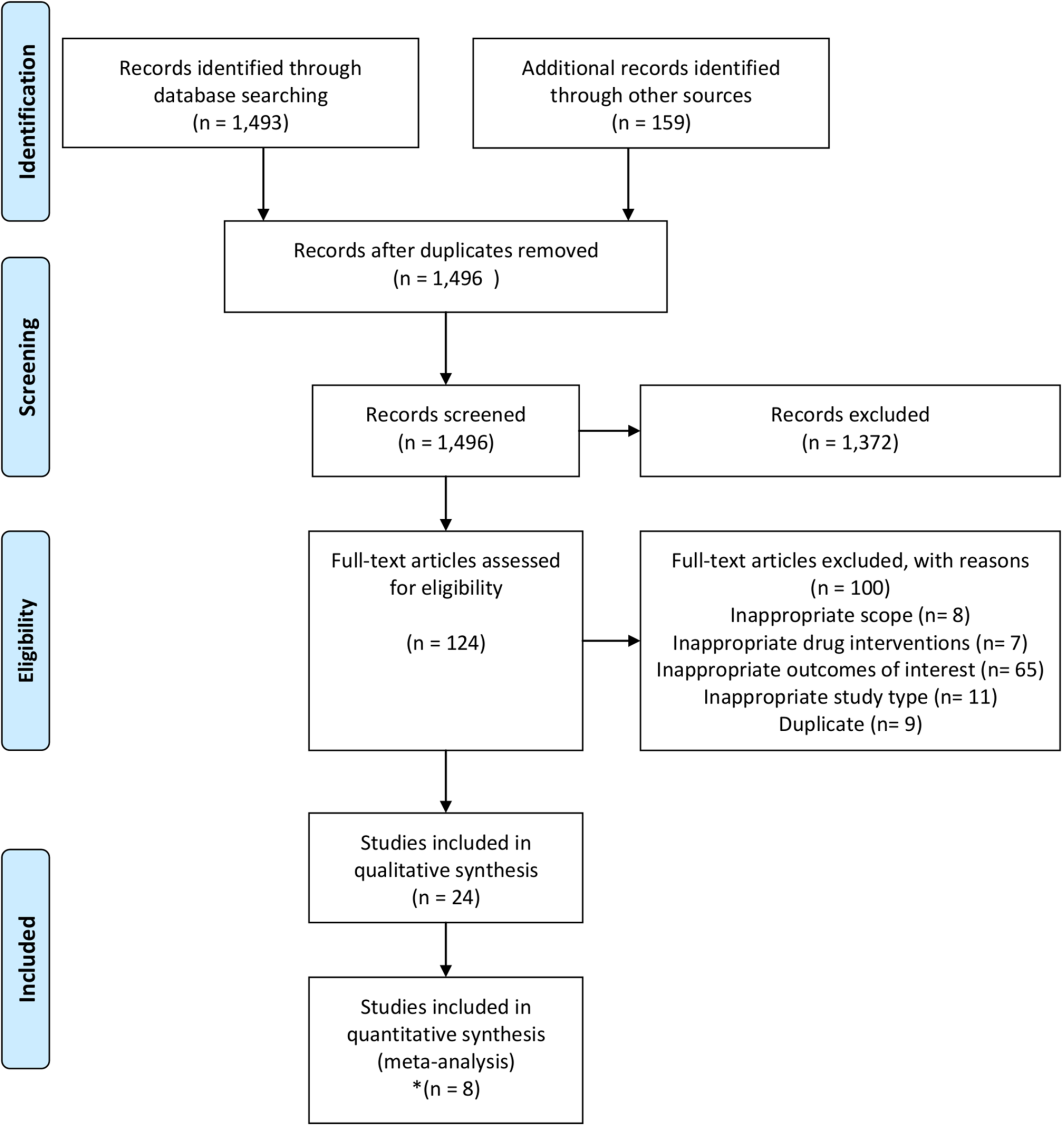
Flow-chart of systematic literature search. ^*^One congress abstract (Gallant et al. 2012) [50] which was presented during the XIX international AIDS conference was replaced by a complete communication that became available at the data-extraction stage (Gallant et al. 2013) [12].

The flow diagram of the systematic review selection process is displayed in Fig 1. The search identified 1,649 titles/abstracts. After the removal of duplicate entries (153 hits), 1,496 titles/abstracts were screened of which 1,372 were excluded according to the pre-specified criteria. The remaining 124 full-text articles were reviewed resulting in a total of 24 articles (22 RTCs and 2 cohort studies)[12;20–42] undergoing data extraction. Among them, six different outcomes were identified. Eighteen studies reported measuring eGFR using different formulae (i.e. Cockcroft–Gault [CG], CKD-Epidemiology [CKD-EPI] or Modification Diet Renal Disease [MDRD] equations), eleven reported renal dysfunction measured by serum/plasma creatinine, two reported proteinuria and one reported any renal symptom. In addition, one study reported serious renal disease and another reported acute renal failure as outcomes. For the meta-analysis, we selected the renal outcomes which were reported in more than two studies to allow the formation of potential networks. This lead to further examination of studies reporting eGFR/creatinine clearance, serum/plasma creatinine and proteinuria. The studies varied greatly with respect to the timing of assessment, the method of measurement, the measured variables and the units. The follow-up period differed between studies. Depending on the studies and the reported renal outcomes, renal function was assessed at 2, 24, 48, 96, or 144 weeks or at varying times if the follow-up duration was not fixed. Furthermore, the change in eGFR was measured by various methods. Six studies estimated GFR using the CG formula, five the MDRD method, five the CKD-EPI formula and five did not specify the method of assessment.

For the quantitative meta-analysis, we decided to focus on eGFR as it is considered the best overall index of kidney function in health and disease.[3] Studies with the same timing of assessment, method of measurement, measured variables and units were included for pooled unadjusted analysis. Therefore, we assessed the change from baseline in GFR at 48 weeks (the most commonly reported timepoint) estimated by CG method (six studies)[12;21;28;31;35;36] and the change from baseline in GFR at 48 weeks estimated by CKD-EPI and MDRD methods (four studies)[25;28;29;31].

**Fig 2:**
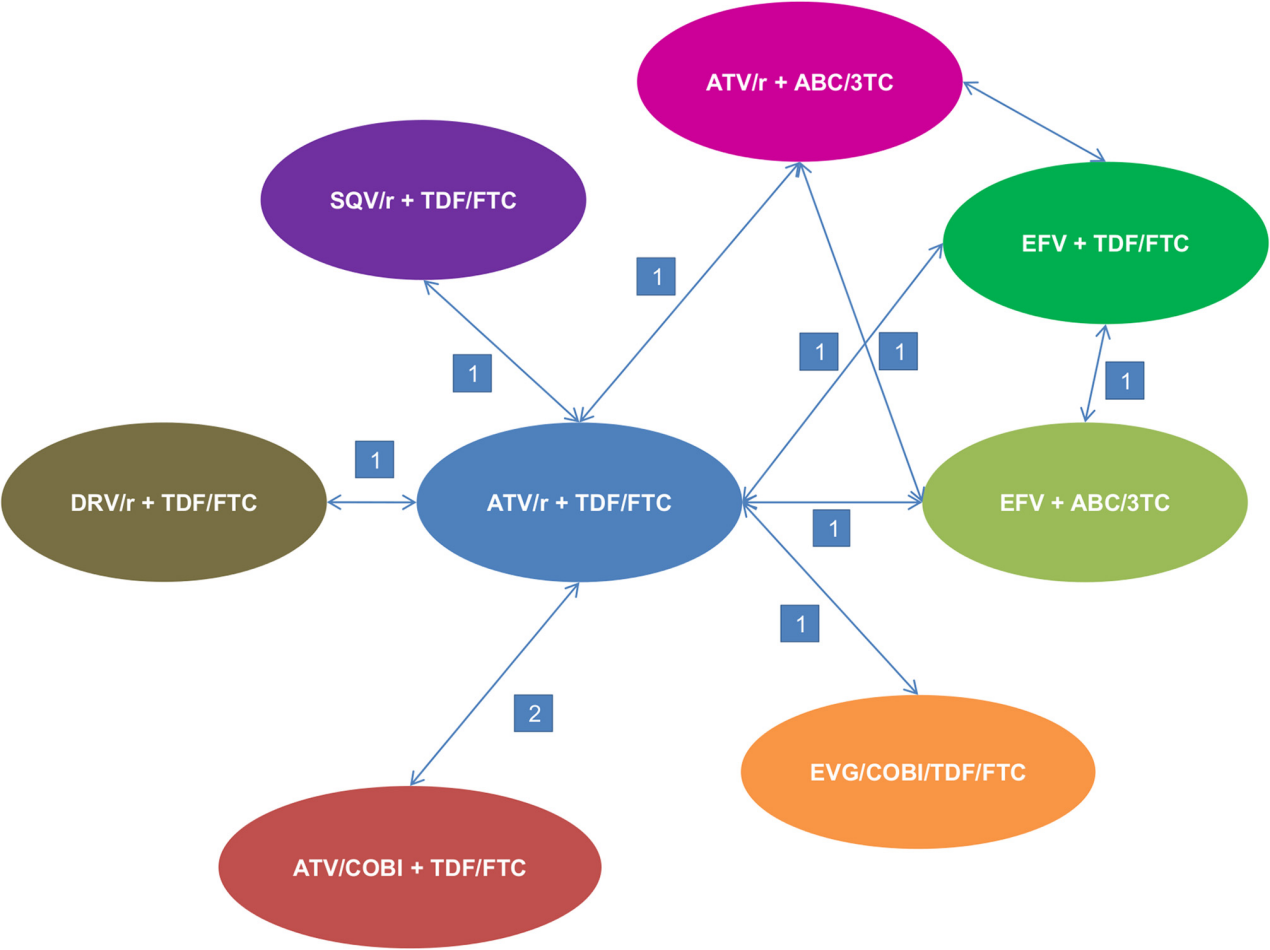
Network diagram—change in eGFR from baseline using the CG method. CG: Cockcroft–Gault; eGFR: estimated Glomerular Filtration Rate. The number on the arrowed line represents the number of studies reported pairwise comparison.

**Fig 3:**
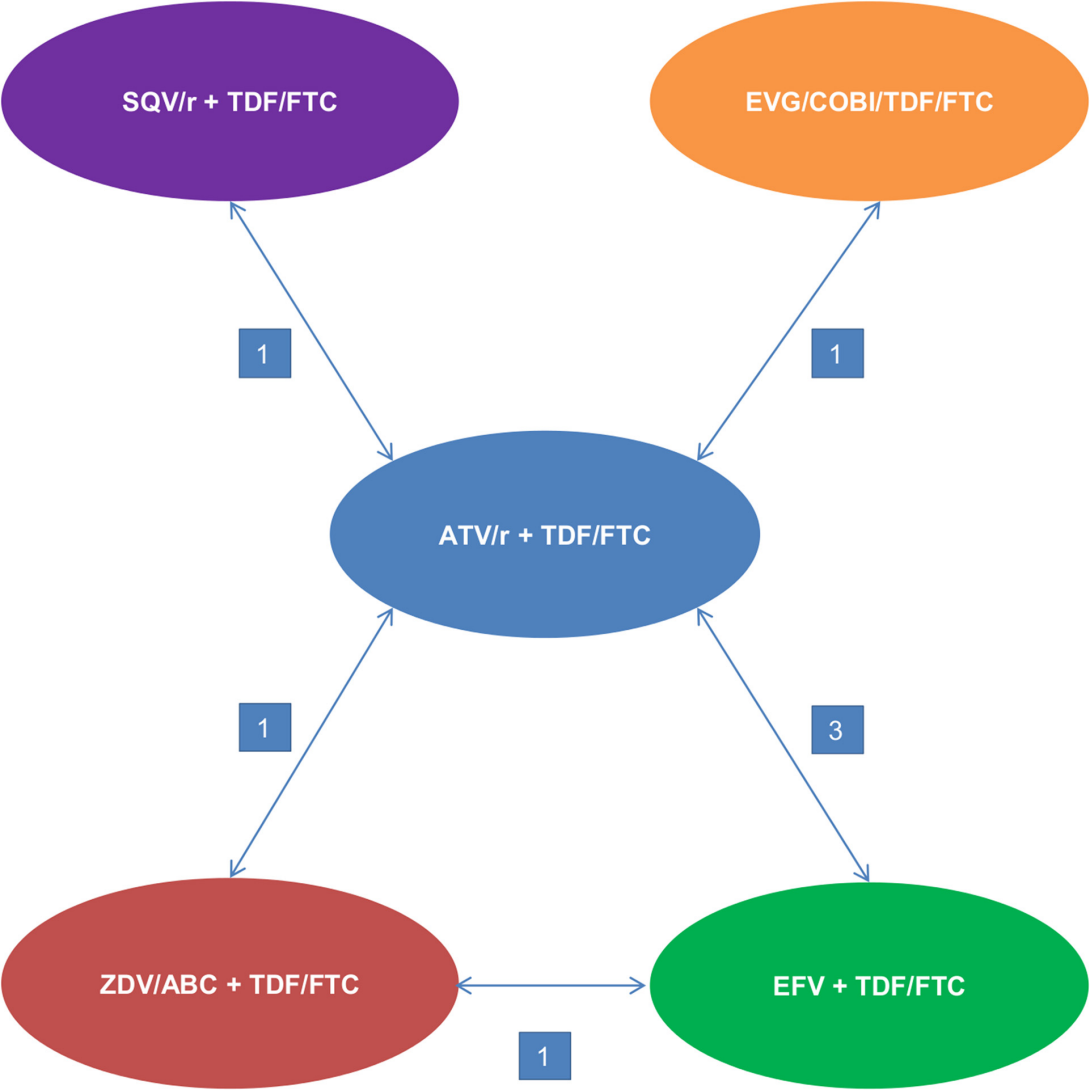
Network diagram—change in eGFR from baseline using the pooled MDRD and CKD-EPI methods. CKD-EPI: Chronic Kidney Disease-Epidemiology; eGFR: estimated Glomerular Filtration Rate; MDRD: Modification Diet Renal Disease. The number on the arrowed line represents the number of studies reported pairwise comparison.

We designed two treatment networks and conducted two different meta-analyses based on either the mean change from baseline in GFR at 48 weeks estimated by CG method (Fig 2) or the change from baseline in GFR at 48 weeks estimated by pooled MDRD and CKD-EPI methods (Fig 3). Patient characteristics and study design of the included studies are presented in Tables 2 and 3. Three ATV regimens and six comparator regimens were represented in the included studies; in total, nine regimens were combined in the first network and six in the second one:

- Atazanavir boosted with ritonavir + Tenofovir/ emtricitabine (ATV/r + TDF/FTC)
- Atazanavir boosted with cobicistat + Tenofovir/ emtricitabine (ATV/cobi + TDF/FTC)
- Atazanavir boosted with ritonavir + Abacavir/ lamivudine (ATV/r + ABC/3TC)
- Elvitegravir/ cobicistat/ tenofovir/ emtricitabine (EVG/cobi/TDF/FTC)
- Efavirenz + Tenofovir/ emtricitabine (EFV + TDF/FTC)
- Efavirenz + Abacavir/ lamivudine (EFV + ABC/3TC)
- Saquinavir + Tenofovir/ emtricitabine (SQV/r + TDF/FTC)
- Darunavir boosted with ritonavir + Tenofovir/ emtricitabine (DRV/r + TDF/FTC)
- Zidovudine + Tenofovir/ emtricitabine (ZDV/ABC + TDF/FTC)

**Table 2:** Study design and patients’ demographic characteristics.

Primary author, publication date	Study type, name	Location	Study recruitment period	Age (years) of participants	Sex (Male (%))	Race/Ethnicity (%)
**Gallant, 2013[12]**	RCT, GS-US-216-0114	International	April 2010—November 2010	Median (SD): 37.5 ^a^ (9.70)^b^	83.0 ^a^	^a^ White: 59.7; Black or African heritage: 18.5; Asian: 11.7; Other: 8.7; Unkown: 0.8; American Indian or Alaska Native: 0.5
**Elion, 2011[35]**	RCT, GS-US-216-0105	United-States	NR	Mean: 35.9 ^a^	91.0 ^a^	^a^ White: 59.4; Black/African heritage: 33.1; Other: 4.9; Asian: 2.6
**DeJesus, 2012[28]**	RCT, GS-236-0103	International	NR	Mean (SD): 38.5 ^a^ (10.16) ^b^	90.0 ^a^	^a^ White: 74.5; Black/African heritage: 16.5; Hispanic/latino: 15.5; Asian: 5.0
**Daar, 2011[36]**	RCT, A5202	United-States and Puerto Rico	September 2005—Novemzber 200	Median (Q1-Q3): 38 (31–45)	83.0	White/non-Hispanic: 40; Black/non-Hispanic: 33; Hispanic: 23; Asian/Pacific Islander: 2; Native American/Alaskan Native: 1; >1 race: 1
**Vrouenraets, 2011[31]**	RCT, BASIC	Multinational	February 2006—June 2007	Mean (SD): 38.4 ^a^ (9.86) ^b^	84.8 ^a^	^a^ Caucasian: 60.2; Black: 27.1; Other: 8.5; Hispanic: 5.3
**Aberg, 2012[21]**	RCT, METABOLIK	NR	NR	Median (range): 35.8 ^a^ (19.0–65.0)	87.1 ^a^	^a^ White: 50.8; Black: 46.1; Asian: 3.1
**Dazo, 2011[25]**	RCT, ALTAIR	NR	NR	Mean (SD): 37.0 (8.9)	78.0	Caucasian: 58; Asian: 33; Hispanic: 5; Black:4
**Albini, 2012[29]**	RCT	Italy	June 2007—April 2009	Mean (SD): 43.66 (11.52)	79.1	Other: 98.9; Black: 1.1

Q1-Q3: Quartile 1-Quartile 3; NR: Non Reported; RCT: Randomized Clinical Trial; SD: Standard Deviation

^a^ Weighted average between the treatment arms

^b^ SD was calculated assuming the samples were independent and normally distributed

**Table 3:** Renal baseline characteristics and treatment regimens of patients.

Primary author, publication date	Treatment arms	Number of patients	eGFR at baseline	Measured variable at 48 weeks (Change from baseline)	Method for the eGFR
**Gallant, 2013[12]**	ATV/r + TDF/FTC	348	NR (inclusion criteria: eGFR ≥ 70 mL/min	Median	CG
	ATV/cobi + TDF/FTC	344	NR (inclusion criteria: eGFR ≥ 70 mL/min	Median	CG
**Elion, 2011[35]**	ATV/r + TDF/FTC	29	122 mL/min	Mean	CG
	ATV/cobi + TDF/FTC	50	117 mL/min	Mean	CG
**DeJesus, 2012[28]**	ATV/r + TDF/FTC	355	115 mL/min	Mean	CG, MDRD
	EVG/cobi/TDF/FTC	353	113 mL/min	Mean	CG, MDRD
**Daar, 2011[36]**	ATV/r + TDF/FTC	394	118 mL/min^†^	Mean	CG^†^
	EFV + TDF/FTC	360	119 mL/min^†^	Mean	CG^†^
	EFV + ABC/3TC	338	119 mL/min^†^	Mean	CG^†^
	ATV/r + ABC/3TC	377	120 mL/min^†^	Mean	CG^†^
**Vrouenraets, 2011[31]**	ATV/r + TDF/FTC	61	112 mL/min	Mean	CG, CKD-EPI
	SQV/r + TDF/FTC	57	110 mL/min	Mean	CG, CKD-EPI
**Aberg, 2012[21]**	ATV/r + TDF/FTC	31	NR (exclusion criteria: CrCl ≤ 50 ml/min/1.73m^2^	Mean	*CG
	DRV/r + TDF/FTC	34	NR (exclusion criteria: CrCl ≤ 50 ml/min/1.73m^2^	Mean	*CG
**Dazo, 2011[25]**	ATV/r + TDF/FTC	99	104 mL/min/1.73m^2^	Mean	CKD-EPI
	EFV + TDF/FTC	100	102 mL/min/1.73m^2^	Mean	CKD-EPI
	ZDV/ABC + TDF/FTC	76	103 mL/min/1.73m^2^	Mean	CKD-EPI
**Albini, 2012[29]**	ATV/r + TDF/FTC	48	100 mL/min/1.73m^2^	Mean	CKD-EPI
	EFV + TDF/FTC	43	100 mL/min/1.73m^2^	Mean	CKD-EPI

CG: Cockcroft–Gault; CKD-EPI: Chronic Kidney Disease-Epidemiology; CrCl: Creatinine Clearance; eGFR: estimated Glomerular Filtration Rate; MDRD: Modification Diet Renal Disease; NR: Not Reported

* Method not specified but deduced from the reported unit (mL/min)

^†^ The values or method was known after having contacted the author

### Heterogeneity assessment

Depending on the trials, patients were eligible for inclusion if they had an eGFR superior or equal to 50 mL/min (Aberg, 2012 and Albini, 2012)[21;29], eGFR superior or equal to 70 mL/min (Gallant, 2013, Dejesus, 2012 and Dazo, 2011)[12;25;28] and eGFR superior or equal to 80 mL/min (Elion, 2011)[35]. While no eligible criteria on renal function were reported in the Vrouenraets and Daar studies, the patients had a generally normal eGFR at baseline (eGFR = 111 mL/min and eGFR = 119 mL/min on average, respectively).[31;36] Potential sources of heterogeneity in the baseline characteristics of the patient populations were assessed within the included studies (Table 2). Studies had similar patient characteristics with respect to median age (35–45), gender distribution, and BMI, when available. Regarding the ethnicity characteristic, it is known that Black people have higher serum-creatinine concentration because of an average higher muscular mass. The number of Black patients ranged from 2.1% (Albini, 2012)[29] to 54.8% (Aberg, 2012)[21] and is a potential source of heterogeneity. An ethnic correction factor is applied to the MDRD and CKD-EPI equations. Using the MDRD formula, such factor would be accurate in Afro-American and African patients with CKD but would be too high for those with an eGFR superior or equal to 60 mL/min per 1.73 m^2^, leading to an underestimation of the prevalence of CKD.[43]

### Meta-analysis results

#### Relative risk of renal impairment at 48 weeks measured with CG

The estimated mean change in eGFR from baseline and their associated SD and SE for each trial are shown in Table 4. The six trials considered in the analysis, all assessing at least one boosted ATV arm, included only treatment-naïve patients with a total of 3,131 patients.[12;21;28;31;35;36]

**Table 4:** Mean change in eGFR from baseline from individual studies (CG method).

Primary author—study name	Treatment dose	Mean change from baseline (mL/min)	SD	SE
**Gallant, 2013[12]—GS-US-216-0114**	ATV/r + TDF/FTC—300/100 + 300/200 mg^¤^	-9.10^*^ (median)	13.64^a^	0.73^c^
	ATV/cobi + TDF/FTC—300/150 + 300/200 mg	-12.90^*^ (median)	12.45^a^	0.67^c^
**Elion, 2011[35]—GS-US-216-0105**	ATV/r + TDF/FTC—300/100 + 300/200 mg	-11.00^**^	17.11^μ^	3.18^c^
	ATV/cobi + TDF/FTC—300/150 + 300/200 mg	-13.00^**^	12.45^μbis^	1.76^c^
**Dejesus, 2012[28]—GS-236-0103**	ATV/r + TDF/FTC—300/100 + 300/200 mg	-9.30^*^	16.00^*^	0.85^c^
	EVG/cobi/TDF/FTC—150/150/300/200 mg	-13.30^*^	14.00^*^	0.75^c^
**Daar, 2011[36]—A5202**	ATV/r + TDF/FTC—300/100 + 300/200 mg	0.50^*^	21.70^†^	1.09^c^
	EFV + TDF/FTC—600 + 300/200 mg	4.70^*^	21.40^†^	1.13^c^
	EFV + ABC/3TC—600 + 600/300 mg	5.80^*^	20.80^†^	1.13^c^
	ATV/r + ABC/3TC—300/100 + 600/300 mg	4.40^*^	23.00^†^	1.18^c^
**Vrouenraets, 2011[31]—BASIC**	ATV/r + TDF/FTC—300/100 + NR mg	-1.00^***^	46.44^b^	5.95^c^
	SQV/r + TDF/FTC—2,000/100 + NR mg	-10.00^***^	37.67^b^	4.99^c^
**Aberg, 2012[21]—METABOLIK**	ATV/r + TDF/FTC—300/100 + 300/200 mg	-0.03^*^	0.24^*^	0.04^c^
	DRV/r + TDF/FTC—800/100 + 300/200 mg	0.00^*^	0.29^*^	0.05^c^

CG: Cockcroft–Gault; eGFR: estimated Glomerular Filtration Rate; NR: Non Reported; SD: Standard Deviation; SE: Standard Error

^*^ Values retrieved from the paper

^**^ Calculated using eGFR at baseline and at 48 weeks

*** Values retrieved graphically and deduced using the difference in the mean change from baseline

^a^ Calculated using the interquartile range and assuming a normal distribution, see Eq (1) in the statistical analysis section

^b^ Calculated using the Eq (2) detailed in the statistical analysis section

^c^ Calculated using the Eq (3) detailed in the statistical analysis section

^μ^ Calculated using the weighted average of the available SD associated with the ATV/r + TDF/FTC arm from Gallant, 2013, Dejesus, 2012 and Daar, 2011 studies

^μbis^ Value estimated by assuming the same SD as the one used in the Gallant, 2013 study for the ATV/cobi + TDF/FTC arm

^†^ Values obtained after contacting the author

^¤^ Dose retrieved from clinicaltrials.gov (NCT01108510)

**Fig 4:**
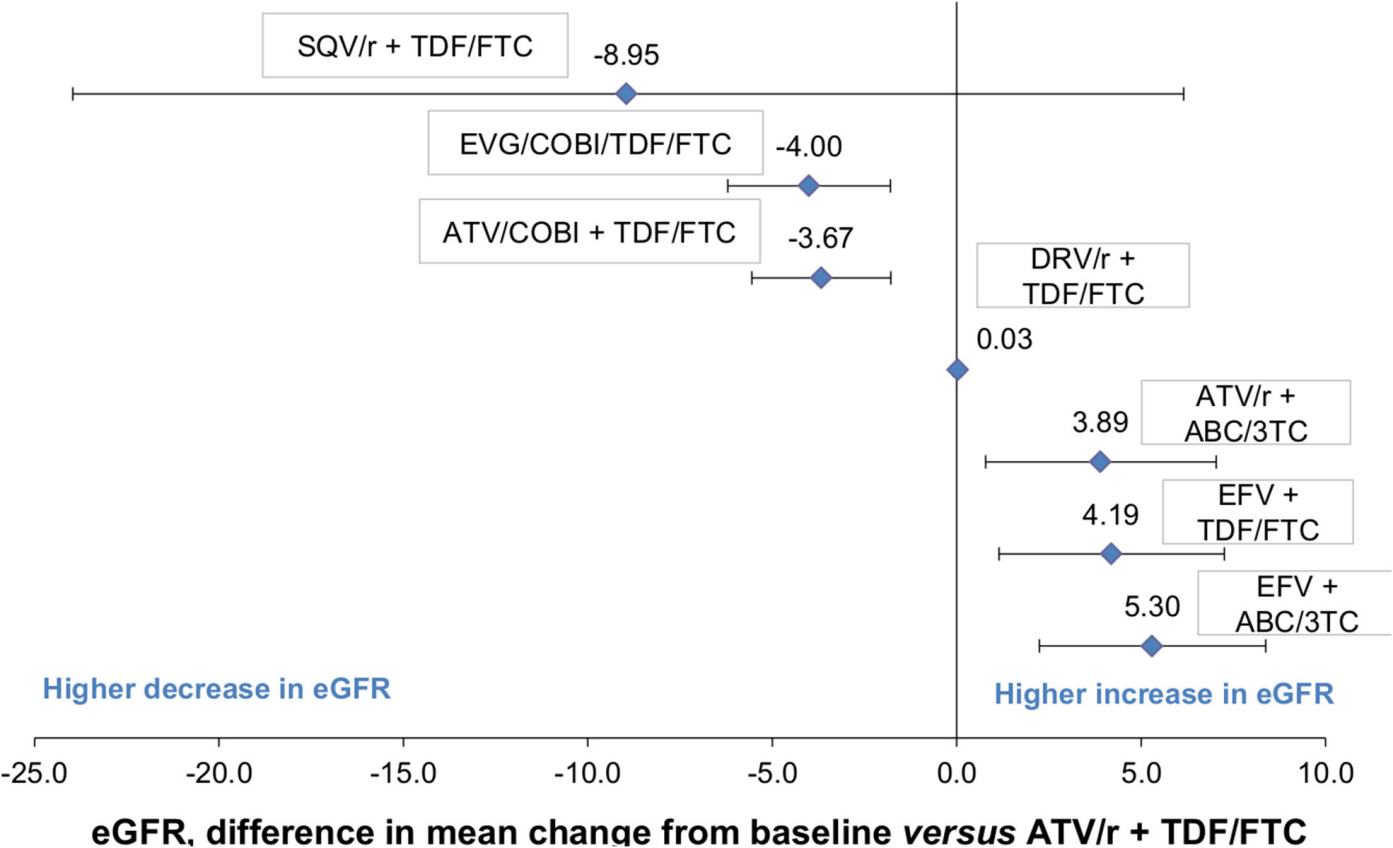
Results from the MTC: Difference in mean change in eGFR from baseline at 48 weeks (using CG method). CG: Cockcroft–Gault; eGFR: estimated Glomerular Filtration Rate; MTC: Mixed-Treatment Comparison.

The estimated difference in the mean change in eGFR from baseline for each comparator treatment and their associated 95% credible intervals *(credible interval in Bayesian statistics are analogous to confidence interval in frequentist statistics)* relative to the reference treatment ATV/r + TDF/FTC is shown in Fig 4 and Table 5. A difference in the mean change in eGFR from baseline less than 0 indicates that one treatment had a higher decrease or lower increase in eGFR in comparison to the reference treatment.

**Table 5:** MTC results providing the difference in mean change in eGFR from baseline compared with ATV/r + TDF/FTC (CG method).

Antiretroviral based regimens	Difference in mean change from baseline	95% CrIs
**ATV/r + TDF/FTC**	1	
**SQV/r + TDF/FTC**	-8.95	[-23.96;6.15]
**EVG/cobi/TDF/FTC**	-4.00	[-6.21;-1.79]
**ATV/cobi + TDF/FTC**	-3.67	[-5.56;-1.79]
**DRV/r + TDF/FTC**	0.03	[-0.09;0.16]
**ATV/r + ABC/3TC**	3.89	[0.78;7.03]
**EFV + TDF/FTC**	4.19	[1.15;7.25]
**EFV + ABC/3TC**	5.30	[2.24;8.38]

95%CrIs: 95% Credible Intervals; CG: Cockcroft–Gault; eGFR: estimated Glomerular Filtration Rate; MTC: Mixed-Treatment Comparison

- With respect to NRTI backbone, when combined with ABC/3TC, ATV/r was associated with a lower decrease in eGFR compared with its combination with TDF/FTC. The difference in the mean change in eGFR between the arms was +3.89, (95%CrI 0.78; 7.03). No difference was shown when considering EFV as a third agent (EFV + ABC/3TC vs. EFV + TDF/FTC, +1.11, 95%CrI -2.04; 4.26).
- With respect to the third agent, when combined with TDF/FTC, ATV/cobi and EVG/cobi were considered equivalent in terms of impact on the mean change in eGFR. No difference was shown in the mean change in eGFR between ATV/r and SQV/r when used with TDF/FTC (-9.00, 95%CrI -23.96; 6.15). Of note, similar eGFR change was observed between ATV/r and EFV when combined with ABC/3TC (+1.41, 95%CrI -1.80; 4.59). On the other hand, ATV/r was associated with a lower increase in eGFR compared to EFV (-4.19, 95%CrI -7.25; -1.15).
- With respect to boosting agent, ATV/r was more likely to have less of a decline in eGFR compared with ATV/cobi, both in combination with TDF/FTC (ATV/r + TDF/FTC *vs*. ATV/cobi + TDF/FTC, +3.67, 95%CrI +1.79; +5.56).

**Fig 5:**
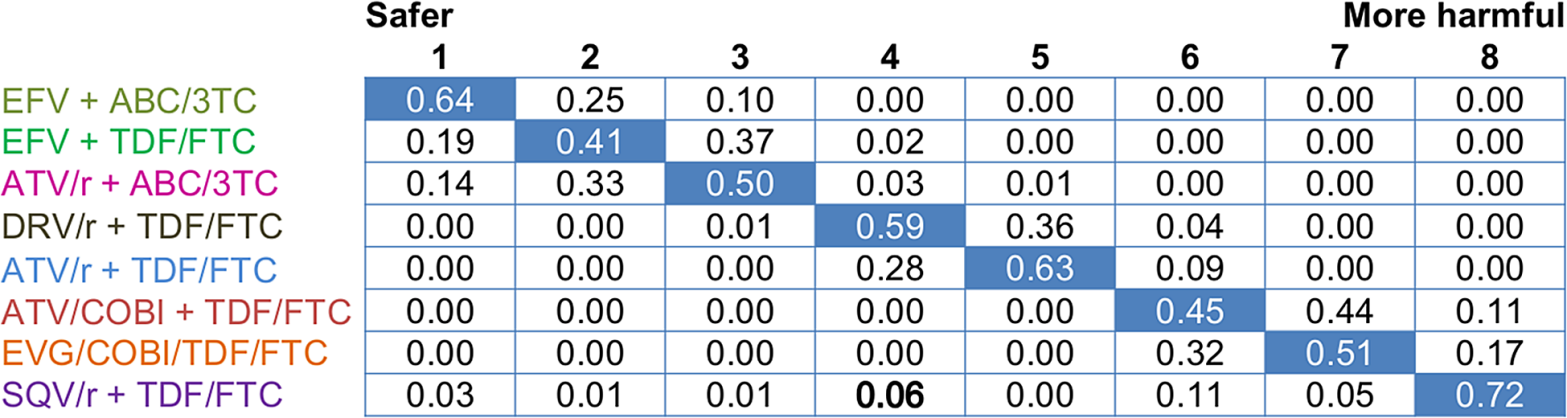
48-week safety ranking on the mean change in eGFR from baseline (assessed with the CG method). CG: Cockcroft–Gault; eGFR: estimated Glomerular Filtration Rate. The values of this double entry table were computed using the rank function in WinBugs 1.4. Each row represents the probability of a treatment occupying different rankings (the blue one being the highest one) and each column represents the probability of different treatments occupying a specific rank (the blue one being the highest one).The table is organised so that the treatment with the highest probability of being ranked first (safest) is placed at the top row and the treatment with the highest probability of begin ranked last (most harmful) is at the bottom.

All treatments were ranked based on their respective probability of being the safest (from Rank 1: “Safer treatment” to Rank 8: “More harmful treatment”) (Fig 5). ATV/r + ABC/3TC was ranked as the third safest treatment with a probability of 0.50, while ATV/r + TDF/FTC and ATV/cobi + TDF/FTC were more likely to impact the GFR score.

#### Relative risk of renal impairment at 48 weeks measured with MDRD and CKD-EPI

The estimated mean change in eGFR from baseline and their associated SD and SE for each trial are shown in Table 6. The four trials considered in the analysis, all assessing at least one boosted ATV arm, included only treatment-naïve patients with a total of 1,192 patients.[25;28;29;31]

**Table 6:** Mean change in eGFR from baseline from individual studies (pooled MDRD and CKD-EPI methods).

Primary author—study name	Treatment—dose	Mean change from baseline (mL/min per 1.73m^2^)	SD	SE
**Dazo, 2011[25]—ALTAIR**	ATV/r + TDF/FTC—300/100 + 300/200 mg^¤^	-4.10^*^	13.71^c^	1.38^d^
	EFV + TDF/FTC—600 + 300/200 mg	1.50^*^	11.99^c^	1.20^d^
	ZDV/ABC + TDF/FTC—250 or 300 + 300/200 mg	1.20^*^	13.34^c^	1.53^d^
**Albini, 2012[29]—NR**	ATV/r + TDF/FTC—300/100 + 300/200 mg	-4.90^*^	13.48^μ^	1.95^c^
	EFV + TDF/FTC—600 + 300/200 mg	1.70^*^	11.99^μbis^	1.83^c^
**Dejesus, 2012[28]—GS-236-0103**	ATV/r + TDF/FTC—300/100 + 300/200 mg	-9.50^*^	13.42^a^	0.71^c^
	EVG/cobi/TDF/FTC—150/150/200/300 mg	-12.70^*^	12.97^a^	0.69^c^
**Vrouenraets, 2011[31]—BASIC**	ATV/r + TDF/FTC—300/100 + NR mg	-6.98^**^	26.70^b^	3.42^c^
	SQV/r + TDF/FTC—2,000/100 + NR mg	-9.20^**^	24.34^b^	3.22^c^

CKD-EPI: Chronic Kidney Disease-Epidemiology; eGFR: estimated Glomerular Filtration Rate; MDRD: Modification Diet Renal Disease; NR: Non Reported; SD: Standard Deviation; SE: Standard Error

^a^ Calculated using the interquartile range and assuming a normal distribution, see Eq (1) in the statistical analysis section

^b^ Calculated using the Eq (2) detailed in the statistical analysis section

^c^ Calculated using the Eq (3) detailed in the statistical analysis section

^d^ Calculated using the Eq (4) detailed in the statistical analysis section

^*^ Values retrieved from the paper

^**^ Calculated using eGFR data at baseline and at 48 weeks, data retrieved graphically;

^μ^ Calculated using the weighted average of the available SD associated with the ATV/r + TDF/FTC arm from Dazo, 2011 and Dejesus, 2012

^μbis^ Value estimated by assuming the same SD as the one used for Dazo, 2011 study for the ATV/cobi + TDF/FTC arm

^¤^ Dose retrieved from clinicaltrials.gov (NCT00335322)

**Fig 6:**
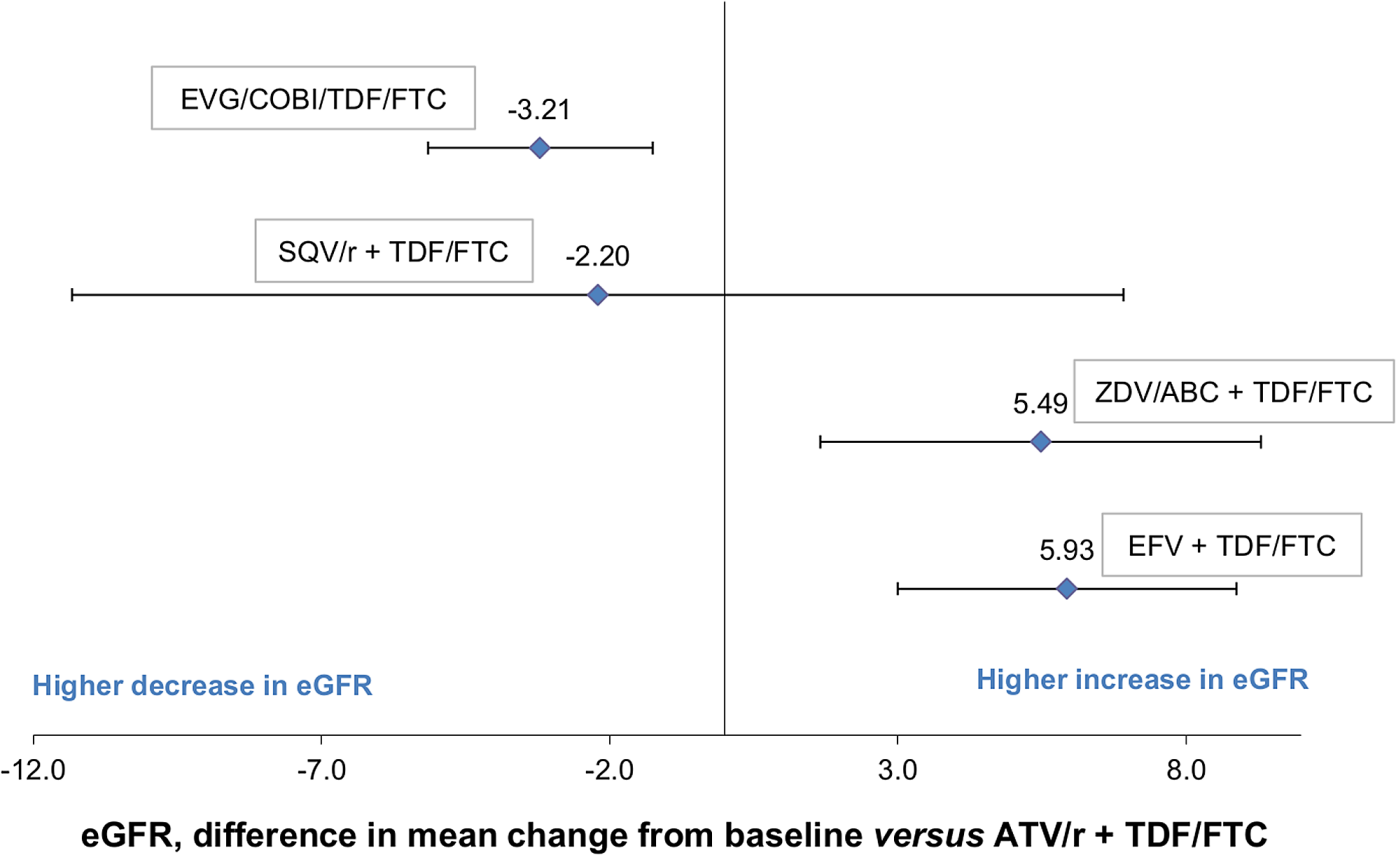
Results from the MTC: Difference in mean change in eGFR from baseline at 48 weeks (using MDRD and CKD-EPI methods). CKD-EPI: Chronic Kidney Disease-Epidemiology; eGFR: estimated Glomerular Filtration Rate; MDRD: Modification Diet Renal Disease; MTC: Mixed-Treatment Comparison.

The estimated difference in the mean change in eGFR from baseline for each active treatment and their associated credible intervals relative to study comparator ATV/r + TDF/FTC is shown in Fig 6 and Table 7. When the differences in the mean change in eGFR from baseline measured with both methods were available from studies that compared the same two active treatments, the results were quite similar with the exception of the SQV/r-containing regimen. Indeed, although eGFR calculated using CG remained stable in the ATV/r arm (mean change in eGFR from baseline = -1.00 mL/min), it decreased when estimated by CKD-EPI (mean change in eGFR from baseline = -6.98 mL/min).

**Table 7:** MTC results providing the difference in mean change in eGFR from baseline compared with ATV/r + TDF/FTC (pooled MDRD and CKD-EPI methods).

Antiretroviral based regimens	Difference in mean change from baseline	95% CrIs
**ATV/r + TDF/FTC**	1	
**EVG/cobi/TDF/FTC**	-3.21	[-5.15;-1.25]
**SQV/r + TDF/FTC**	-2.20	[-11.33;-6.92]
**ZDV/ABC + TDF/FTC**	5.49	[1.66;9.31]
**EFV + TDF/FTC**	5.93	[3.00;8.88]

95%CrIs: 95% Credible Intervals; CKD-EPI: Chronic Kidney Disease-Epidemiology; eGFR: estimated Glomerular Filtration Rate; MDRD: Modification Diet Renal Disease; MTC: Mixed-Treatment Comparison

ATV/r + TDF/FTC was associated with higher decline in eGFR compared to ZDV/ABC + TDF/FTC and EFV + TDF/FTC (-5.49, 95%CrI -9.31;-1.66 and -5.93, 95%CrI -8.88; -3.00). However, there was a lower decrease in eGFR between patients receiving ATV/r + TDF/FTC compared to patients receiving EVG/cobi + TDF/FTC, (+3.21, 95%CI 1.25; 5.1) and no difference was shown with SQV/r (-2.20, 95%CrI -11.33; 6.92).

**Fig 7:**
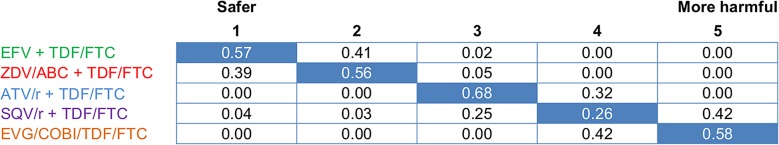
48-week safety ranking on the mean change in eGFR from baseline (assessed with the MDRD and CKD-EPI methods). CKD-EPI: Chronic Kidney Disease-Epidemiology; eGFR: estimated Glomerular Filtration Rate; MDRD: Modification Diet Renal Disease. The values of this double entry table were computed using the rank function in WinBugs 1.4. Each row represents the probability of a treatment occupying different rankings (the blue one being the highest one) and each column represents the probability of different treatments occupying a specific rank (the blue one being the highest one).The table is organised so that the treatment with the highest probability of being ranked first (safest) is placed at the top row and the treatment with the highest probability of begin ranked last (most harmful) is at the bottom.

Fig 7 shows that ATV/r + TDF/FTC occupied an intermediate rank in terms of change in eGFR.

## Discussion

With significant reductions in mortality and aging of the HIV population in the era of ART, long-term complications of HIV infection and associated therapies have become increasingly important. This includes the specific impact antiretroviral agents may have on renal function which was the subject of this analysis. The potential renal toxicity of ART is probably under-appreciated in patients with HIV since renal safety is rarely reported as a primary or secondary outcome in RCTs assessing ART efficacy. To the best of our knowledge this is the first meta-analysis using MTC that attempts to formally estimate the effect of different ART on eGFR.

In the analysis where CG was the method of assessment of eGFR, the MTC results showed that with respect to NRTI backbone, TDF containing combinations showed a more pronounced decline in eGFR versus non-TDF containing regimens. ATV/r + ABC/3TC showed an increase in eGFR rather than a decline when compared with ATV/r + TDF/FTC. This observation suggests that the factor influencing eGFR decline may not be not ATV/r itself but rather TDF/FTC and/or its potential interaction with the third agent or booster. Our findings appear in agreement with an extensive body of literature that point to the direct effect TDF has on renal function, in particular at the tubular level.[44] RTV, which is often co-administered in combination with TDF, has been described as an inhibitor of the active tubular secretion of TDF mediated by the MRP-2 transporter. As a result, this inhibition can lead to intracellular accumulation of TDF within the tubules, which in turn can lead to increased tubular toxicity.[12;45] A recent observational study, the DAD (Data collection on Adverse events of Anti-HIV Drugs) cohort showed that cumulative exposure to TDF (median follow-up duration of 4.5 years) was independently associated with increased rates of progression to eGFR equal or below 70 mL/min.[46]

The relative impact on eGFR seemed not only to be driven by TDF-containing regimen but also by the choice of third agent, especially boosted-PIs. When combined with TDF/FTC, ATV/r as well as ATV/cobi were more likely to be associated with a decrease in eGFR contrary to EFV which led to an increase in eGFR (CG method). When estimated with the pooled MDRD and CKD-EPI methods, ATV/r + TDF/FTC was associated with a higher decline in eGFR compared to ZDV/ABC + TDF/FTC and EFV + TDF/FTC on the one hand and, but on the other hand, was associated with a lower decrease in eGFR compared to EVG/cobi + TDF/FTC. Our results were consistent with conclusions from observational studies. In recent years, evidence from existing large cohorts has shown that ATV/r may be associated with a decline in eGFR compared to other ART.[22;38;46] The EuroSIDA cohort study used renal impairment as a primary outcome (eGFR<60 ml/min per 1.73 m^2^) and found that patients on boosted-PI, mostly with ATV/r, were more likely to develop a decline in eGFR over time compared with EFV.[38] The Swiss HIV cohort study reported a lower decrease in eGFR (estimated with the MDRD formula) for EFV + TDF/FTC compared to ATV/r + TDF/FTC at 24 weeks (difference in eGFR: -7.6 ml/min per 1.73m^2^; 95% CI -11.8 to -3.4).[22] From our analysis, the same trend was observed with an estimated difference in eGFR of -5.93 (95% CI—9.3 to -1.15) at 48 weeks. The DAD cohort data showed that cumulative exposure to ATV/r (median follow-up duration of 4.5 years) was independently associated with increased rates of progression to an eGFR inferior or equal to 70 mL/min, while unboosted ATV was not.[46]

The impact a booster may have on ATV in the ART regimen is important to consider, especially as it relates to its impact on TDF metabolism. In the analysis, using RTV compared to cobicistat as a boosting agent for ATV co-administered with TDF/FTC regimen was associated with less of a decline in eGFR from baseline over 48 weeks (CG method). This lower decline in eGFR associated with RTV might have been expected since it is known that the RTV/TDF interaction leading to tubular toxicity does occur after 48 weeks. As for cobicistat, it would affect eGFR through a different mechanism than RTV. In-vitro studies suggest that cobicistat may increase serum creatinine levels and thus reduce eGFR, through inhibition of proximal renal tubular cell transporters (e.g. MATE-1).[46] However, the potential for renal drug interactions between cobicistat and TDF appears to be low with in vitro and ex vivo data suggesting that the transport mechanism responsible for the tubular secretion of TDF may be minimally affected by cobicistat.[47;48]. In vivo, the renal safety results of a study comparing EVG/cobi/TDF/FTC to ATV/r + TDF/FTC showed that cobicistat- containing regimen appears to lead to a 10–15 mL decline in eGFR within the first month of administration, followed by a plateau from week 18 to 24 with no further change over time (up to 144 weeks).[15] The ongoing phase III trial, study 114, comparing ATV/cobi versus ATV/r in combination with TDF/FTC has reported oucomes at 48 weeks and should further assess the long-term renal safety of ATV/cobi (144 week results awaited).[12] Thus, the importance of distinguishing true declines in eGFR from the possible artifactual decreases in eGFR caused by cobicistat remains to be elucidated. In this analysis we tried to identify the effect of the choice of a NRTI backbone, third agent or booster, all other things being equal; however, this study does not provide information on the safety profile of each agent taken separately. The standard of care of HIV therapy includes a combination of several ARTs, typically three or four, thus the role of individual ART drugs on renal impairment cannot be assessed in patient trials. However, preclinical pharmacology and pharmacokinetics studies focusing on the effects of HIV agents on nephrotoxicity and on renal transporters may help elucidate the mechanism behind renal dysfunction.

There are some limitations that need to be taken into consideration when interpreting the results of this MTC. First, this study highlights the heterogeneity in reporting renal outcomes in the literature; it seems that there is no clear consensus on how to consistently define renal outcomes and on how to best measure renal function in clinical practice. This considerable heterogeneity was an obstacle to our pooled analysis of study results. Sixteen studies identified in our search strategy did not report the most common renal outcomes and thus were excluded at the heterogeneity assessment. For instance, we excluded the study from Mills et *al*. 2012 because the change in eGFR from baseline was reported at a different and less common timing of assessment (96 weeks).[39] Furthermore, the ARIES study, which included patients receiving an unboosted ATV-containing regimen, was not included due to its study design. Indeed, ART-naïve subjects were enrolled in a single arm trial to receive ATV/r + ABC/3TC before being randomized at week 36, between maintaining or discontinuing RTV, to evaluate the effect of unboosted ATV on renal function up to week 144. Thus the impact on renal function of regimens containing unboosted ATV was not assessed in our analysis. However, in the analysis of the ARIES study, no statistically significant decline in eGFR was noted from baseline to week 144 between ATV or ATV/r, each in combination with ABC/3TC.

Second, our study focused on change in eGFR from baseline at 48 weeks and did not look at the long-term safety profile of the different treatment regimens of interest. This choice was constrained by the limited number of studies in the literature reporting renal safety data beyond 48 weeks. Furthermore, there is no evidence whether the observed impact on the eGFR within 48 weeks could be extrapolated beyond that period. Among the studies included in the quantitative analysis, two studies (Dazo et *al*. 2011 and Daar et *al*. 2012) reported changes in eGFR up to 96 weeks.[25;36] The Daar study showed that the increase from baseline in eGFR in patients receiving ATV/r plus ABC/3TC at week 48 was maintained at week 96. The Daar and Dazo studies reported a continuous decline in eGFR between 48 and 96 weeks in patients receiving ATV/r + TDF/FTC. Despite of being curative, the aim of ART drugs is to control disease progression through long-term inhibition of HIV replication. HIV-infected patients will commit to taking lifetime ART drugs and their long-term impact on renal function would need further investigation.

Another limitation of our study is that the results may not be generalizable to a broad HIV population. In the selected studies included in our analysis, women were under-represented. In addition elderly as well as obese patients or patients with lower lean body weight due to cachexia were not enrolled. Depending on the equations used to assess glomerular filtration, these equations do not provide accurate estimates of true renal function in these subgroups of patients. Furthermore, in some studies using CG formula to estimate the GFR, Afro-Americans and Africans representated a non-negligible proportion of the enrolled population (25–55%).[12;21;28;31;35;36] It is well known that the CG equation does not adjust for race leading to a potential source of heterogeneity in terms of eGFR measurement. Also, the different formulae have not been validated for the HIV-infected population; therefore, there is no preferred equation to use.[49] Given the eligibility criteria used in the trials included in the analyses, our study population had all preserved renal function at baseline. Our results may not be considered as accurate for patients with impaired renal function before starting therapy or for patients at risk of a greater decline, such as diabetic or hypertensive patients.

Finally, the limited number of studies available for inclusion makes it difficult to assess the between-study heterogeneity and differentiating this from the treatment effect. Given the paucity of the data included in the network, any additional information could have a significant impact on the results. The current results need to be interpreted with caution. We recommend updating this analysis when new evidence and treatment modalities become available.

## Conclusion

Overall, ATV/r or ATV/cobi administered in combination with TDF/FTC appeared to lead to a decrease in eGFR from baseline ranging from -0.03 to -13.00 mL/min over 48 weeks. Such small decline is unlikely to be of clinical significance, especially in patients with a preserved renal function at baseline (eGFR superior or equal to 100 mL/min), but these results may shed light on the clinician decision for patients with known renal impairment or comorbid diseases affecting renal function such as diabetes mellitus, hypertension or hepatitis C virus co-infection.

ATV/r combined with a non-TDF backbone (i.e. ABC/3TC) led to an increase in eGFR, supporting the idea that the co-adminstration of RTV and TDF in a combination may be the factors leading to eGFR decline.

Large cohorts such as the DAD suggest that unboosted ATV does not significantly impact eGFR; unfortunately it could not be assessed in this meta-analysis.[46]

Because individual ART drugs are not prescribed to patients in “real life”, biological experiments could be valuable to assess the renal toxicity of unboosted agents as ATV, COBI, RTV or TDF.

This systematic review and meta-analysis intended to clarify the impact of various ATV-containing regimens on renal function in patients with HIV. This study did not demonstrate evidence of decreased renal function related specifically to ATV. The clinical impact of these findings remain difficult to interpret, especially with the scarcity of evidence and the short follow-up period of assessment that does not cover for the full treatment duration. Long-term data could be collected through registries and inform on the risk decline of eGFR over time.

When more renal safety data expressed in standard units in HIV patients become available, results from an updated meta-analysis could guide clinicians on the favourable initial ARV treatment regimen to prescribe.

## Supporting Information

S1 Table Search terms and results. (PDF)

S2 Table Current formula used to estimate patients glomerular filtration rate. Age, in years; eC_Cr_: estimated Creatinine Clearance; S_Cr_: Sserum Creatinine in μmol/L; Weight in kg; SU: Serum Urea in mmol/L; Alb = Serum Albumin in g/L; k is 0.7 for females and 0.9 for males; a is -0.329 for females and -0.411 for males; min indicates the minimum of S_Cr_/k or 1; max indicates the maximum of S_Cr_/k or 1.
(TIF)

S3 Table PRISMA checklist. (DOC)
