# Supplementary material for: Systematic Literature Review and Meta-Analysis of Renal Function in Human Immunodeficiency Virus (HIV)-Infected Patients Treated with Atazanavir (ATV)-Based Regimens
Source: PLoS One. 2015 May 4;10(5):e0124666. doi: 10.1371/journal.pone.0124666 (PMC4418798; doi:10.1371/journal.pone.0124666)
Supplement: S1 Table — (PDF) [file pone.0124666.s001.pdf]

Search #	Topic	Detailed Strategy	Number of Hits
**PubMed**			
#1	**Disease area**	"HIV"[Mesh] OR "Acquired Immunodeficiency Syndrome"[Mesh] OR human immunodeficiency virus OR AIDS OR acquired immunodeficiency syndrome	342,959
#2	**Atazanavir**	"atazanavir" [Supplementary Concept] or atazanavir or reyataz	1,030
#3	**Exclusion**	pilot study or "children"	765,991
#4	**Results**	#1 AND #2 NOT #3	938
#5	**Total results with limits**	Limit #4: From 2000, Humans, English	**883**
**EMBASE**			
#1	**Disease area**	*Human immunodeficiency virus/ or *acquired immune deficiency syndrome/ or HIV.hw.	105,581
#2	**Atazanavir**	exp atazanavir plus ritonavir/ or exp atazanavir/ or atazanavir.mp. or reyataz.mp.	4,931
#3	**Exclusion**	exp pilot study/ or exp child/ or pilot study.mp. or child.mp. or children.mp.	2,149,206
#4	**Results**	#1 AND #2 NOT #3	692
#5	**Total results with limits**	Limit #4: From 2000, Humans, English	**493**
**Cochrane Library**			
#1	**Disease area (Limits: 2000-2012)**	Human immunodeficiency virus[Mesh]OR "Acquired Immunodeficiency Syndrome"[Mesh] or human immunodeficiency virus or AIDS or acquired immunodeficiency syndrome	15,464
#2	**Atazanavir**	atazanavir or reyataz	137
#3	**Exclusion**	"pilot study" or "children"	57,714
#4	**Total results with limits**	(#1 AND #2) NOT (#3)	**95**
**CRD (DARE + HTA + NHS-EED)**			
#1	**Disease area (any fields)**	human immunodeficiency virus or AIDS or acquired immunodeficiency syndrome or HIV	1,468
#2	**Atazanavir**	atazanavir or reyataz	20
#3	**Total results**	#1 AND #2	**19**
**ICAAC**			
#1	**ICAAC 2011**	atazanavir	3
#2	**ICAAC 2012**	atazanavir	10
#3	**Total results**	#1 OR #2	**13**
**IDSA**			
#1	**IDSA 2011**	atazanavir	5
#2	**IDSA 2012**	atazanavir	4
#3	**Total results**	#1 OR #2	9
**CROI**			
#1	**CROI 2011**	atazanavir	27
#2	**CROI 2012**	atazanavir	29
#3	**CROI 2013**	atazanavir	30
#4	**Total results**	#1 OR #2 OR #3	86
**IAS/AIDS**			
#1	**IAS 2011**	atazanavir	31
#2	**AIDS 2012**	atazanavir	20
#3	**Total results**	#1 OR #2	51
**Total Hits**			1649
**Duplicates**			153
**Total Hits (deduplicated)**			**1496**
