# Supplementary figures and images for: Systematic Literature Review and Meta-Analysis of Renal Function in Human Immunodeficiency Virus (HIV)-Infected Patients Treated with Atazanavir (ATV)-Based Regimens
Source: PLoS One. 2015 May 4;10(5):e0124666. doi: 10.1371/journal.pone.0124666 (PMC4418798; doi:10.1371/journal.pone.0124666)

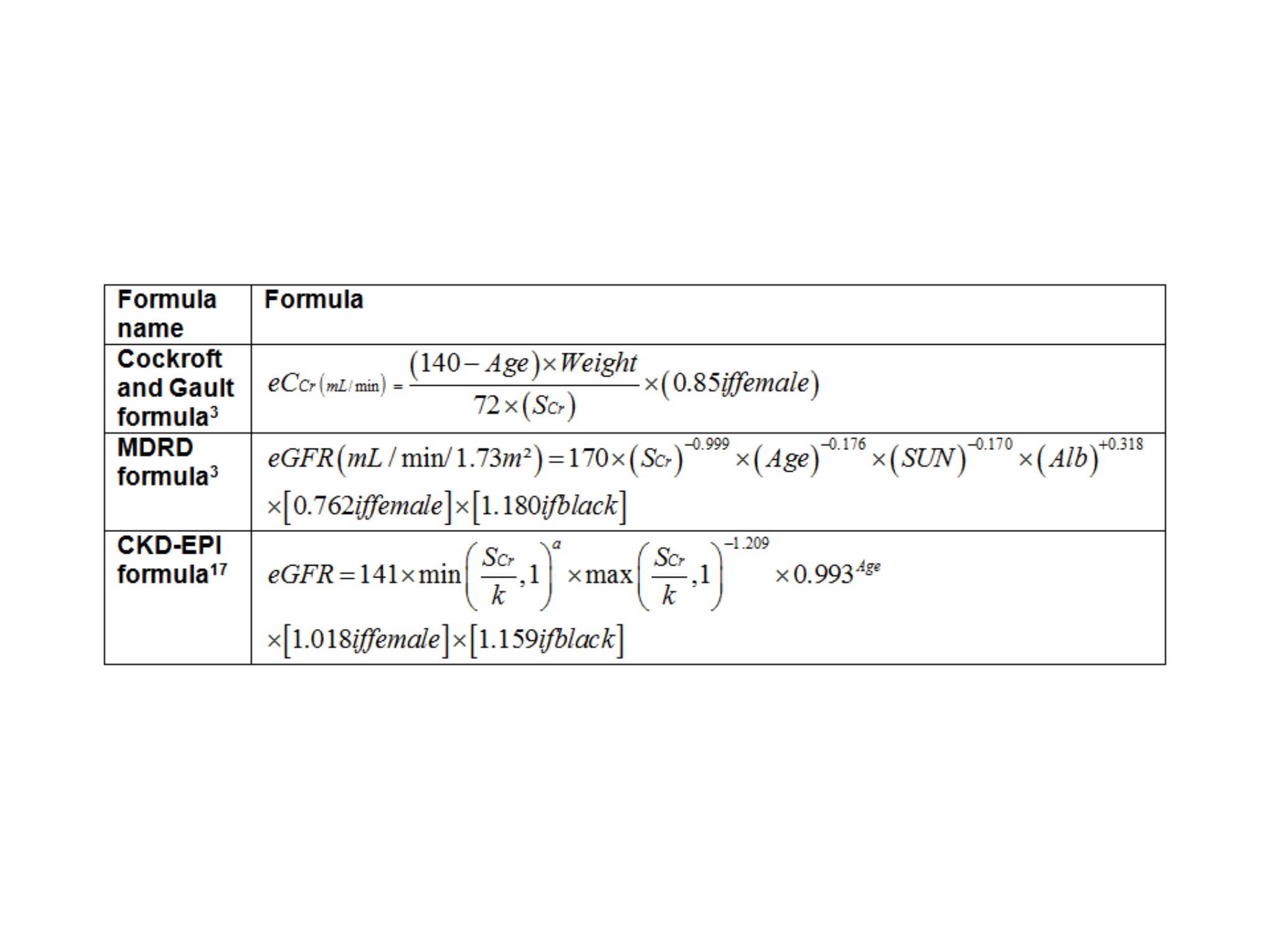

Supplement: S2 Table — Age, in years; eCCr: estimated Creatinine Clearance; SCr: Sserum Creatinine in μmol/L; Weight in kg; SU: Serum Urea in mmol/L; Alb = Serum Albumin in g/L; k is 0.7 for females and 0.9 for males; a is -0.329 for females and -0.411 for males; min indicates the minimum of SCr/k or 1; max indicates the maximum of SCr/k or 1. (TIF) [file pone.0124666.s002.tif]
